# How Smart Is It to Go to Bed with the Phone? The Impact of Short-Wavelength Light and Affective States on Sleep and Circadian Rhythms

**DOI:** 10.3390/clockssleep3040040

**Published:** 2021-10-28

**Authors:** Sarah R. Schmid, Christopher Höhn, Kathrin Bothe, Christina P. Plamberger, Monika Angerer, Belinda Pletzer, Kerstin Hoedlmoser

**Affiliations:** 1 Laboratory for Sleep, Cognition and Consciousness Research, Department of Psychology, University of Salzburg, 5020 Salzburg, Austria; sarahrebeca.schmid@plus.ac.at (S.R.S.); christopher.hoehn@plus.ac.at (C.H.); kathrin.bothe@plus.ac.at (K.B.); christina.plamberger@plus.ac.at (C.P.P.); monika.angerer@plus.ac.at (M.A.); 2 Centre for Cognitive Neuroscience Salzburg (CCNS), University of Salzburg, 5020 Salzburg, Austria; belinda.pletzer@plus.ac.at

**Keywords:** light exposure, short-wavelength light, blue-light filter, sleepiness, slow wave sleep, slow wave activity, melatonin, cortisol, distal-proximal skin temperature gradient, affective states

## Abstract

Previously, we presented our preliminary results (N = 14) investigating the effects of short-wavelength light from a smartphone during the evening on sleep and circadian rhythms (Höhn et al., 2021). Here, we now demonstrate our full sample (N = 33 men), where polysomnography and body temperature were recorded during three experimental nights and subjects read for 90 min on a smartphone with or without a filter or from a book. Cortisol, melatonin and affectivity were assessed before and after sleep. These results confirm our earlier findings, indicating reduced slow-wave-sleep and -activity in the first night quarter after reading on the smartphone without a filter. The same was true for the cortisol-awakening-response. Although subjective sleepiness was not affected, the evening melatonin increase was attenuated in both smartphone conditions. Accordingly, the distal-proximal skin temperature gradient increased less after short-wavelength light exposure than after reading a book. Interestingly, we could unravel within this full dataset that higher positive affectivity in the evening predicted better subjective but not objective sleep quality. Our results show disruptive consequences of short-wavelength light for sleep and circadian rhythmicity with a partially attenuating effect of blue-light filters. Furthermore, affective states influence subjective sleep quality and should be considered, whenever investigating sleep and circadian rhythms.

## 1. Introduction

Human sleep is highly vulnerable to external (i.e., ambient light) and internal (i.e., affective states) influences. Indeed, natural light is the most important “Zeitgeber”, that synchronizes our “inner clock” to the geophysical time, i.e., adapting our sleep-wake cycle to a 24 h rhythm [1,2,3]. With the development of electricity, artificial light became a new important “Zeitgeber” that influences our circadian rhythm. Given the growing use of screen-based media devices during the evening over the last decade [4], the impact of light on circadian rhythmicity and sleep is increasingly discussed. Smartphones and other electronic devices are equipped with light-emitting-diodes (i.e., LEDs), which emit a high proportion of short-wavelength light [5], peaking in wavelengths around 460 nm [6,7]. This is of special interest, as so-called intrinsically photosensitive ganglion cells in the retina (ipRGCs) are expressing the photopigment melanopsin, which is highly sensitive to light of short wavelengths between 446 and 484 nm [8]. Thereby, short-wavelength light influences the suprachiasmatic nuclei (SCN; i.e., the circadian pacemaker), which receive environmental information from the retina [9,10]. In turn, the SCN control different autonomic functions, as well as behavior via projections to different brain regions [11]. As summarized by Tosini and colleagues, this non-image forming framework around melanopsin stimulation, specifically by short-wavelength light, is powerful in affecting a variety of physiological and circadian functions and can even affect ocular health [12]. Therefore, it is of importance to discuss potential health hazards due to the circadian effects together with positive aspects of an exposure to light of different wavelengths at different times of the day [13]. Accordingly, recent evidence shows that short-wavelength light emitting devices are able to reduce subjective sleepiness in the evening and lead to higher levels of sleepiness in the morning [14]. Bright light in general is even discussed and successfully applied as a method to decrease sleepiness in shift-workers during the night [15]. Interestingly, blue-light-blocking glasses were able to attenuate such light-induced effects [16].

On a biological level, it has been shown that short-wavelength light exposure in the evening initially suppresses the secretion of the hormone melatonin [14] and leads to a delayed increase during exposure [6]. More specifically, the secretion of melatonin is controlled by the pineal gland [17] which receives input from the SCN. The SCN in turn receive information from the pineal gland by means of melatonin secretion, which can increase or attenuate the sleepiness [17,18]. In the evening, melatonin rises exponentially followed by a rapid decline in core body temperature [19] through a decrease in heat production and an increase in heat loss due to distal vasodilation of the vessels. This thermoregulatory cascade is indirectly measured by the distal-proximal gradient (DPG; i.e., difference between proximal and distal skin temperature), which serves as a proxy for the core body temperature. A rapid rise in the DPG (i.e., increasing vasodilation of the vessels leading to heat loss) is identified as the best predictor for a short sleep onset latency among other circadian parameters (cf., Kräuchi and Wirz-Justice [20]). With respect to light effects, it has been shown that short-wavelength light exposure (460 nm) leads to a DPG decrease 2 h after the light exposure. This effect is not visible for longer wavelength light (540 nm) [21].

Besides projecting to the pineal gland, the SCN are also connected to the pituitary gland, which controls the release of cortisol. The cortisol secretion follows the circadian signal, characterized by a low concentration during the day and during the first half of the night [22]. In the second half of the night, the cortisol level slowly rises and results in a strong “cortisol awakening response” (CAR). This is characterized by a rapid increase in cortisol within 30 min to 60 min after awakening [23]. The CAR reflects the hypothalamic-pituitary-adrenal (HPA) axis activity in response to the transition from sleep to wakefulness [24]. Regarding the impact of light on cortisol secretion, 1 h of bright light (414 photopic lux) in the morning 5 min after awakening compared to 1 h of dim light (<2 photopic lux) elicited a higher CAR [25], whereas 1 h of bright light with 800 photopic lux compared to 0 photopic lux in the evening did not affect evening cortisol levels [26]. However, both studies did not investigate the impact of evening light exposure on morning cortisol secretion.

With regard to sleep, light also influences slow wave sleep (SWS). After reading for 30 min on a tablet compared to reading a book, slow electroencephalographic (EEG) activity dynamics within the delta frequency range (i.e., 0.50–3.99 Hz) were delayed by approximately 30 min. Accordingly, delta power was reduced 60 to 105 min after sleep onset [27]. Furthermore, Muench and colleagues [7] found that 2 h of short-wavelength light exposure (460 nm) compared to longer wavelength light exposure (540 nm) or to darkness slightly reduced slow wave activity (SWA; 0.75–4.5 Hz) in the first NREM-cycle and led to a SWA rebound in the third NREM cycle. Additionally, they found a reduction for SWS in both light conditions compared to darkness. However, recent studies show that susceptibility to light exposure differs between individuals [3] and is affected by age [28].

Besides external influences, sleep is also highly affected by psychological variables (e.g., anxiety [29]). In this regard, mental disorders are frequently accompanied by sleep disturbances and poor sleep quality [30]. Even non-pathologically relevant affective states influence sleep quality [31]. Accordingly, individuals with high levels of positive affectivity (PA) in combination with low levels of negative affectivity (NA) showed the highest self-rated sleep quality, compared to the three other affective groups defined by the remaining combinations of high/low PA and NA (i.e., low PA and low NA, low PA and high NA, high PA and high NA). However, Ong and colleagues [32] concluded in their review that PA has a positive impact on subjective sleep quality independently of the severity of NA. Besides subjective sleep quality, objective measures of sleep and their relation to affective states have only been assessed in a few studies: McCrae and colleagues [33] assessed the influence of objective and subjective sleep quality on subsequent affective states. They found that next-day PA was higher after nights of over-average subjective sleep quality and below-average wake time (subjectively rated) but was not related to objective sleep measures. However, it seems logical that the mood in the morning is prone to distort the retroactive rating of sleep quality.

Our study was conducted in a standardized environment under real-life conditions (i.e., no artificially extended exposure times and no dark adaptation phase). In a within-subject design, 33 participants read for 90 min either (1) on a smartphone without or (2) with a blue-light filter or (3) from a printed book. We hypothesized that reading on a smartphone without a filter reduces evening subjective sleepiness, suppresses the evening melatonin increase, reduces distal vasodilation during the night (i.e., lowers the DPG) and leads to a less pronounced CAR the next morning. Concerning sleep parameters, we expected a reduction in early SWS and early SWA. Using a blue-light filter during reading was expected to reduce these negative effects, while reading on printed material served as a control condition. Furthermore, we hypothesized that PA but not NA in the evening predicted self-rated sleep quality and that this relationship would not emerge for objective sleep measures serving as indices for good sleep quality (i.e., sleep onset latency, sleep efficiency, wake time after initial sleep onset).

## 2. Results

In order to test the hypotheses above, repeated measure ANOVAs with follow-up pairwise comparisons, multiple linear regression models and linear mixed-effects models were calculated when assumptions were met. Before analyses, extreme values (more than three times the interquartile range away from the median) were excluded from parametric analyses separately for each statistical test. In case of violation of the assumptions that were still present after exclusion of extreme values, non-parametric Friedman tests and Wilcoxon signed-rank tests were performed including valid data of all participants. Level of significance was set to *p* < 0.05 (two-sided); *p*-values between 0.05 and 0.10 were denoted as trend. In order to counteract multiple comparisons, Bonferroni correction was used. *P*-values that were higher than 0.10 after correction are marked with a cross (†).

### 2.1. Subjective Sleepiness

**Figure 1:**
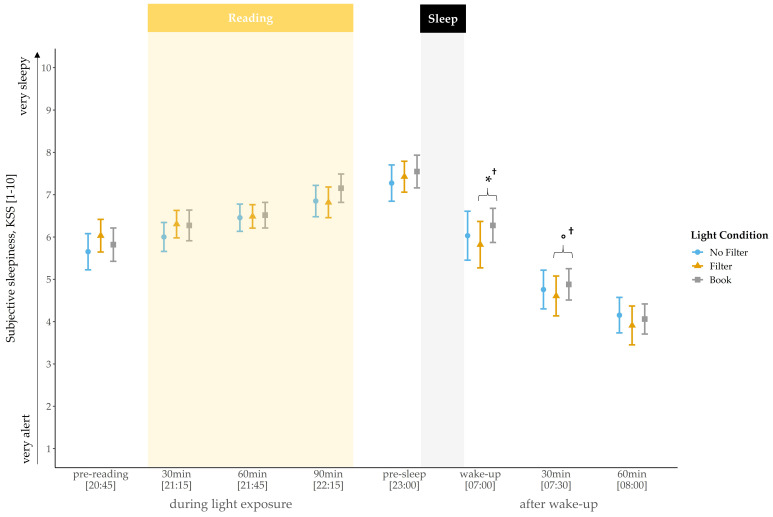
Trajectory of subjective sleepiness (mean and 95% confidence intervals). Subjects were significantly more tired at awakening and by trend more tired 30 min post-awakening after reading a book compared to reading on a smartphone with a filter. Yellow background = light exposure (reading session); gray background = lights turned off (sleep). *: *p* ≤ 0.05; °: *p* < 0.10; †: *p*_ad__j_. > 0.10.

Self-rated sleepiness steadily increased during the evening and showed a rapid decline in the morning across all conditions (Figure 1). At awakening, a statistical trend indicated a main effect for light condition (*χ*^2^(2) = 4.69, *p* = 0.096, *W* = 0.07). Exploratory follow-up comparisons showed that subjects felt less tired at awakening after reading on a smartphone with a filter compared to reading a book on the preceding evening (*z*(N = 33) = −2.12, *p* = 0.034^†^, *r* = 0.37). The main effect for light condition vanished 30 min after awakening. Nevertheless, exploratory follow-up comparisons still indicated a trend for lower sleepiness in the “filter” compared to the “book” condition (*z*(N = 33) = −1.67, *p* = 0.095^†^, *r* = 0.29). No main effect for condition was found for sleepiness ratings at baseline (i.e., before light exposure; *p* = 0.151).

### 2.2. Cortisol and Melatonin

**Figure 2:**
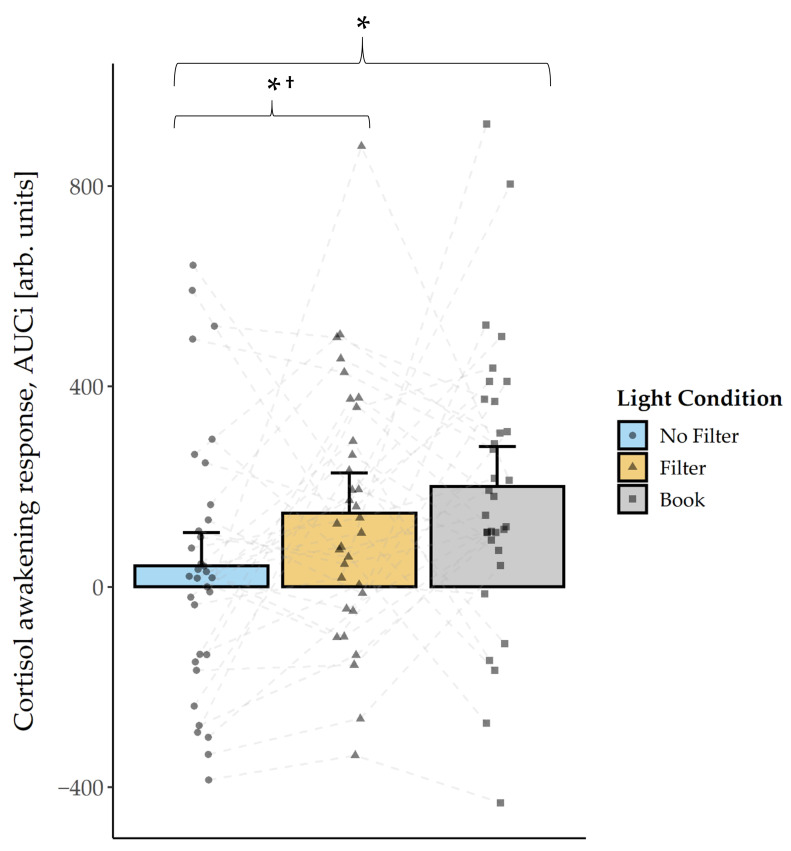
Cortisol awakening response (mean and 95% confidence intervals). The AUCi was calculated for three time points in the morning, i.e., at awakening as well as 30 min and 60 min after awakening. AUCi was significantly smaller in the “no filter” compared to both other light conditions. The dashed lines connect the measurements from each subject. *: *p* ≤ 0.05; †: *p*_ad__j_. > 0.10.

Friedman tests for cortisol concentration did not indicate significant differences between the light conditions in the evening (all *p* > 0.226). Also in the morning, no main condition effect was found for cortisol concentration (all *p* > 0.233). However, explorative post-hoc pairwise comparisons indicated, by trend, a higher cortisol level at wake-up in the “no filter” compared to the “book” condition (*z*(N = 33) = 1.74, *p* = 0.081^†^, *r* = 0.30). Furthermore, 30 min after awakening the pattern changed and cortisol concentration was, by trend, higher in the “book” compared to the “filter” condition (*z*(N = 33) = 1.80, *p* = 0.073^†^, *r* = 0.31), as well as compared to the “no filter” condition (*z*(N = 33) = 1.87, *p* = 0.062^†^, *r* = 0.33). In order to operationalize the cortisol awakening response, we calculated the cortisol AUCi (i.e., cortisol output with respect to the increase) and found a main effect for the factor condition (*F*(2,64) = 4.67, *p* = 0.013, *η^2^* = 0.13). Follow-up pairwise comparisons indicated a smaller cortisol AUCi in the “no filter” compared to the “filter” *(t*(32) = −2.10, *p* = 0.043^†^, *d* = −0.37) and compared to the “book” (*t*(32) = −3.23, *p* = 0.003, *d* = −0.56) condition (Figure 2). Additionally, we compared the raw cortisol increase within 30 min after awakening between the conditions. The statistical analysis indicated a significant main effect for the factor condition (*F*(2,64) = 6.17, *p* = 0.004, *η^2^* = 0.16). Follow-up pairwise comparisons revealed a similar pattern to the AUCi with a weaker cortisol increase after reading on a smartphone without a filter compared to reading on a smartphone with a filter (*t*(32) = −2.84, *p* = 0.008, *d* = −0.49) and compared to reading a book (*t*(32) = −3.10, *p* = 0.004, *d* = −0.54).

**Figure 3:**
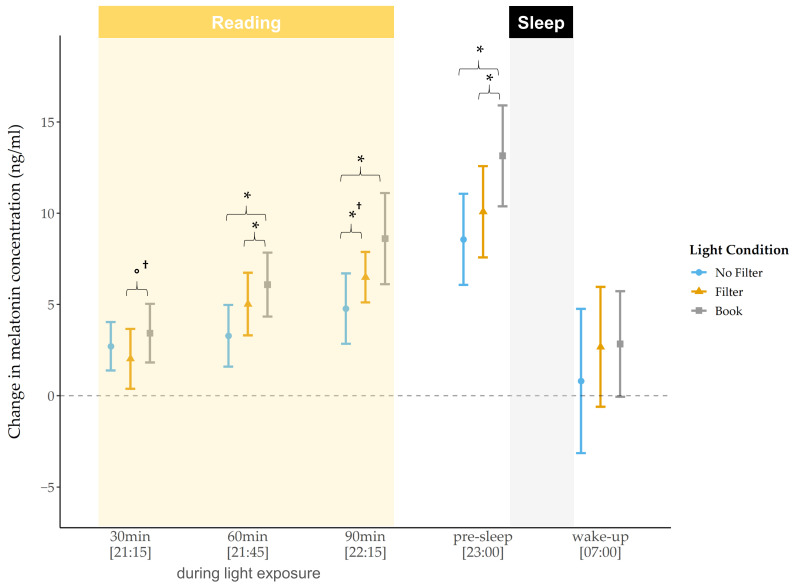
Trajectory of baseline corrected salivary melatonin concentration (mean and 95% confidence intervals). Melatonin increased, by trend, less in the “filter” compared to the “book” condition after 30 min and significantly less after 60 min of light exposure and at bedtime. Melatonin increased significantly less in the “no filter” compared to the “book” condition starting after 60 min of light exposure and persisted until bedtime. Yellow background = light exposure (reading session); gray background = lights turned off (sleep). *: *p* ≤ 0.05; °: *p* < 0.10; †: *p*_ad__j_. > 0.10.

For the raw melatonin concentration levels, we did not find a significant main effect for the factor condition, neither in the evening nor in the morning. However, exploratory post-hoc pairwise comparisons showed a significantly lower melatonin concentration after 30 min of light exposure in the “filter” compared to the “no filter” condition (*z*(N = 33) = −2.44, *p* = 0.015, *r* = 0.42). At bedtime, melatonin concentration was lower in the “filter” condition compared to the “book” condition *(z*(N = 33) = −2.06, *p* = 0.040^†^*, r* = 0.36). Furthermore, we found that differences between the “no filter” and the “filter” condition were already present before light exposure (*z*(N = 33) = −2.06, *p* = 0.039^†^*, r* = 0.36), i.e., the melatonin level was lower in the “no filter” compared to the “filter” condition before any experimental manipulation. After baseline correction (i.e., melatonin concentration “pre-light exposure” were subtracted from melatonin concentration levels at the respective later time points), differences in the melatonin increase between the light conditions became visible (Figure 3). After 30 min and 60 min of light exposure no main effects for light condition could be revealed, but exploratory follow-up comparisons already indicated a trend for a lower melatonin increase in the “filter” compared to the “book” condition (*z*(N = 33) = −1.75, *p* = 0.080^†^*, r* = 0.30) after 30 min of light exposure. Additionally, a significantly lower increase after 60 min of light exposure in the “filter” (*z*(N = 33) = −2.14, *p* = 0.033, *r* = 0.37) and in the “no filter” (*z*(N = 33) = −2.30, *p* = 0.021, *r* = 0.40) compared to the “book” condition was present. Furthermore, we found a significant main effect for condition regarding the melatonin increase after 90 min (*χ^2^* (2) = 9.26, *p* = 0.010, *W* = 0.14) and at bedtime (*χ^2^* (2) = 9.18, *p* = 0.010, *W* = 0.14). Post-hoc comparisons indicated a significantly lower increase in the “no filter” compared to the “filter” (*z*(N *= 33*) = −2.04, *p* = 0.042^†^*, r* = 0.36) as well as compared to the “book” condition (*z*(N = 33) = −2.78, *p* = 0.005, *r* = 0.48) after 90 min of light exposure. At bedtime, the melatonin increase relative to pre-reading was still significantly lower in the “no filter” compared to the “book” condition (*z*(N = 33) = −2.80, *p* = 0.005, *r* = 0.49) and differed between the “filter” and the “book” condition with a lower increase in the “filter” condition (*z*(N = 33) = −2.13, *p* = 0.033, *r* = 0.37). For the melatonin AUCi in the evening, we found no significant main effect for condition (*χ*^2^(2) = 4.42, *p* = 0.109, *W* = 0.07). However, exploratory post-hoc comparisons indicated that the melatonin AUCi was significantly smaller in the “no filter” compared to the “book” condition (*z*(N = 33) = −2.60, *p* = 0.009, *r* = 0.45) and tended to be smaller in the “filter” than in the “book” condition (*z*(N = 33) = −1.90, *p* = 0.057^†^, *r* = 0.33).

**Figure 4:**
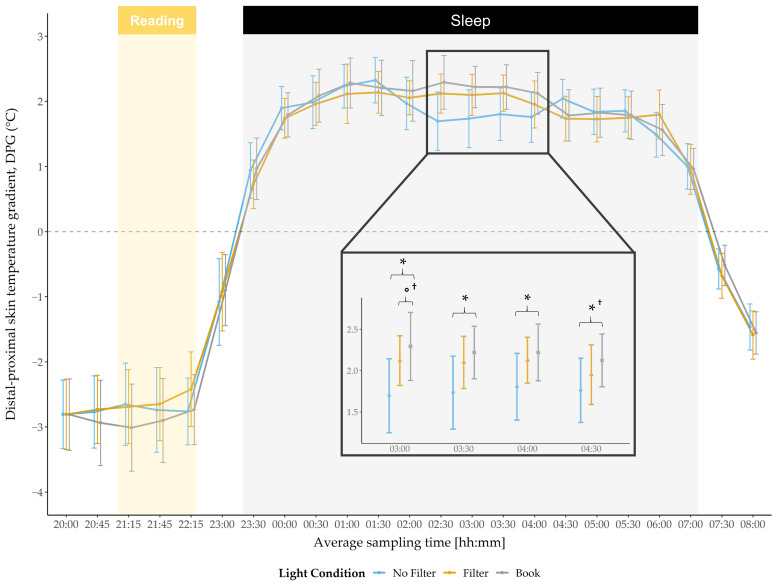
Trajectory of the distal-proximal gradient (mean and 95% confidence intervals). DPG was significantly lower in the “no filter” condition compared to the “book” condition from 3:00 to 4:30 and by trend lower in the “filter” condition compared to the “book” condition at 3:00. Yellow background = light exposure (reading session); gray background = lights turned off (sleep). *: *p* ≤ 0.05; °: *p* < 0.10; †: *p*_ad__j_. > 0.10.

Results regarding the DPG indicated no main effect for condition during the evening or during the morning (Figure 4). However, a significant main condition effect was found during the night at 03:00 (*F*(2,58) = 3.70, *p* = 0.031, *η2 =* 0.11) and by trend at 03:30 (*F*(2,58) = 3.09, *p* = 0.053, *η2 =* 0.10), as well as at 04:00 (*F*(2,58) = 2.67, *p* = 0.078, *η2* = 0.084). Post-hoc pairwise comparisons indicated that the DPG was lower after reading on a smartphone without a filter compared to reading a book at 03:00 (*t*(29) = −2.54, *p* = 0.017, *d* = 1.29), 03:30 (*t*(29) = −2.35, *p* = 0.026, *d =* 1.13), 04:00 (*t*(29) = −2.40, *p* = 0.023, *d =* 0.95) and at 04:30 (*t*(29) = −2.14, *p* = 0.041^†^, *d =* 0.95; explorative analyses). After reading on a smartphone with a filter, the DPG was, by trend, lower compared to reading a book at 03:00 (*t*(29) = −2.01, *p* = 0.054^†^, *d* = 1.16).

### 2.3. Sleep Architecture

Results of the non-parametric Friedman tests for the sleep parameters in the three conditions are shown in Table 1.

The results indicated a main effect for the factor condition assessing the total sleep time (TST), sleep efficiency (SEFF), wake after sleep onset (WASO) and the awakening index. Post-hoc pairwise comparisons showed that participants slept in total significantly less in the “no filter” (*z*(N = 32) = −2.96, *p* = 0.003, *r* = 0.52) and in the “filter” (*z*(N = 32) = −3.55, *p* < 0.001, *r* = 0.63) condition than in the “book” condition. Regarding SEFF, pairwise comparisons indicated that subjects showed a lower sleep efficiency in the “no filter” (*z*(N = 32) = −2.25, *p* = 0.026, *r* = 0.39) and in the “filter” (*z*(N = 32) = −3.32, *p* = 0.001, *r* = 0.59) condition compared to the “book” condition. The same pattern also emerged regarding WASO; participants showed a longer wake time during the night after initial sleep onset in the “no filter” (*z*(N = 32) = 2.06, *p* = 0.039^†^*, r* = 0.36) and in the “filter” (*z*(N = 32) = 2.73, *p* = 0.006, *r* = 0.48) condition than in the “book” condition. Lastly, these results are also resembled in the awakening index scores, where subjects showed more awakenings per hour in the “no filter” (*z*(N = 32) = 2.65, *p* = 0.008, *r* = 0.47) and “filter” condition (*z*(N = 32) = 2.47, *p* = 0.014, *r* = 0.44) than in the book condition (Table 1).

### 2.4. Slow Wave Sleep and Slow Wave Activity

**Figure 5:**
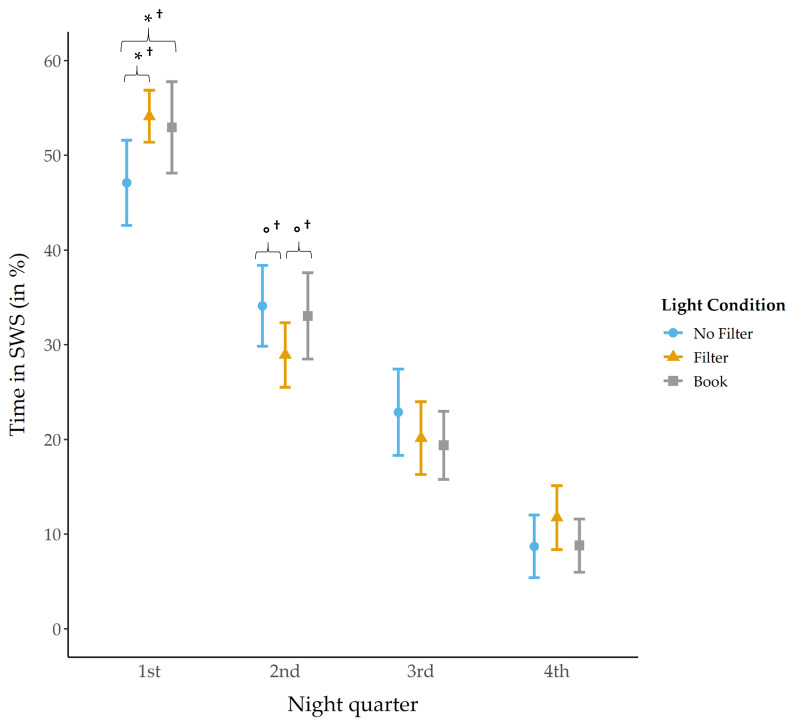
Time in SWS (mean and 95% confidence intervals). The amount of SWS in the first night quarter was significantly reduced in the “no filter” condition as compared to the “filter” and “book” condition. In the second night quarter, the amount of SWS was significantly reduced in the “filter” condition compared to the “no filter” and by trend compared to the “book” condition. *: *p* ≤ 0.05; °: *p* < 0.10; †: *p*_ad__j_. > 0.10.

Time spent in SWS was analyzed separately for each night quarter (Figure 5) and for the whole night.

We did not find a main effect for the factor condition during the first night quarter. However, post-hoc exploratory comparisons showed that subjects spent significantly less time in SWS during the first night quarter after reading without a filter as compared to reading with a filter (*z*(N = 32) = −1.99, *p* = 0.046 ^†^, *r* = 0.35) and as compared to reading from a book (*z*(N = 32) = −1.96, *p* = 0.050^†^*, r* = 0.35). In the second night quarter, again no main condition effect was found for time spent in SWS. However, exploratory post-hoc comparisons indicated that SWS was by trend reduced in the “filter” compared to the “no filter” (*z*(N = 32) = −1.92, *p* = 0.055 ^†^*, r* = 0.34) and also compared to the “book” (*z*(N = 32) = −1.78, *p* = 0.075 ^†^*, r* = 0.31) condition. Concerning the whole night, also no main condition effect was found for time spent in SWS (Table 1).

**Figure 6:**
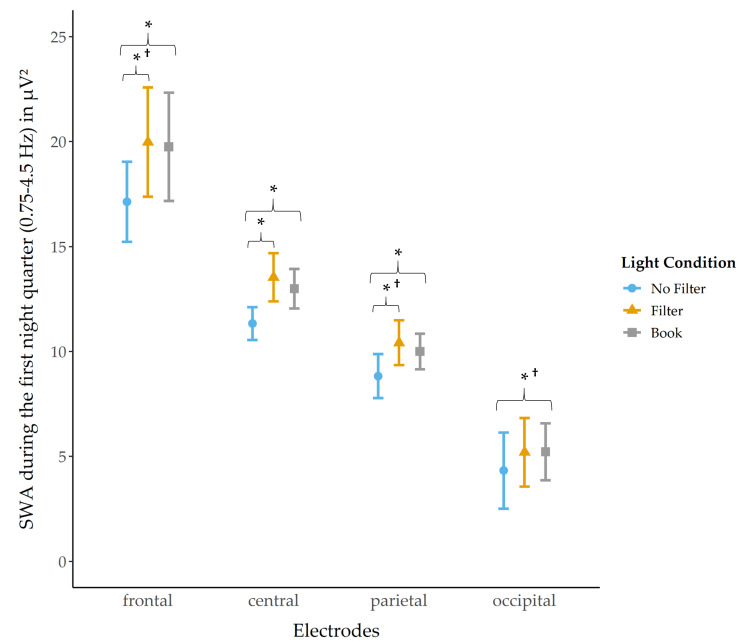
SWA in the first night quarter (mean and 95% confidence intervals). SWA was significantly reduced in the “no filter” compared to the “book” condition at frontal, central, parietal and occipital electrodes. Additionally, SWA was significantly reduced in the “no filter” compared to the “filter” condition at frontal, central and parietal derivations. *: *p* ≤ 0.05; †: *p*_ad__j_. > 0.10.

Analyses for the amount of SWA were separately computed for each night quarter and for the whole night (Supplementary Table S1). SWA during the whole night was highest at the frontal electrodes and declined over central and parietal to occipital regions (all *p* < 0.001; Supplementary Figure S1). A significant main effect for condition was found for the first night quarter at frontal (*χ*^2^ (2) = 7.31, *p* = 0.026, *W* = 0.11), central (*χ*^2^ (2) = 9.81, *p* = 0.007, *W* = 0.15) and parietal (*χ*^2^ (2) = 9.19, *p* = 0.010, *W* = 0.14) derivations. Post-hoc pairwise comparisons revealed that SWA was significantly reduced after reading on a smartphone without a filter at frontal sites as compared to the “filter” *z*(N = 32) = −2.09, *p* = 0.036 ^†^, *r* = 0.37) and “book” condition (*z*(N = 32) = −2.56, *p* = 0.010, *r* = 0.45). At central and parietal sites, the same pattern was observed (central: “no filter” vs. “filter: *z*(N = 32) = −2.51, *p* = 0.012, *r* = 0.44 and “no filter” vs. “book” *z*(N = 32) = −2.54, *p* = 0.011, *r* = 0.45; parietal: “no filter” vs. “filter”: *z*(N = 32) = −1.98, *p* = 0.047 ^†^, *r* = 0.35 and “no filter” vs. “book”: *z*(N = 32) = −2.38, *p* = 0.018, *r* = 0.42). At occipital derivations no main condition effect was found but explorative follow-up comparison indicated that SWA was reduced in the “no filter” compared to the “book” (*z*(N = 32) = −2.08, *p* = 0.038 ^†^, *r* = 0.37) condition (Figure 6).

Explorative analyses regarding the second, third and fourth night quarter showed no further main condition effect. However, explorative post-hoc pairwise comparisons revealed that SWA was, by trend, reduced in the “no filter” compared to the “book” condition in the third night quarter at central (*z*(N = 32) = −1.76, *p* = 0.079 ^†^, *r* = 0.31) and parietal electrodes (*z*(N = 32) = −1.78, *p* = 0.076 ^†^, *r* = 0.31).

Regarding the whole night (Supplementary Figure S1), we examined SWA at frontal, central, parietal and occipital sites and found a significant main effect for condition at frontal derivations (*χ*^2^ (2) = 10.19, *p* = 0.006, *W* = 0.16). Post-hoc pairwise comparisons indicated a significantly reduced SWA in the “no filter” compared to the “filter” (*z*(N = 32) = −2.64, *p* = 0.08, *r* = 0.47) and “book” condition (*z*(N = 32) = −2.02, *p* = 0.043 ^†^, *r* = 0.36). At central derivations, only a trend effect for condition was present (*χ*^2^ (2) = 5.69, *p* = 0.058, *W* = 0.09). However, explorative post-hoc comparisons showed that SWA at central derivations was significantly lower in the “no filter” condition compared to the “filter” (*z*(N = 32) = −2.41, *p* = 0.016, *r* = 0.43) and by trend lower compared to the “book” condition (*z*(N = 32) = −1.74, *p* = 0.082 ^†^, *r* = 0.31). At parietal derivations we found again a significant main effect for condition (*χ*^2^ (2) = 8.31, *p* = 0.016, *W* = 0.13) with, by trend, less SWA in the “no filter” compared to the “filter” condition (*z*(N = 32) = −1.89, *p* = 0.059 ^†^, *r* = 0.33). No significant main effect was found for occipital electrodes. However, explorative post-hoc comparisons indicated again by trend lower SWA in the “no filter” compared to the “filter” condition (*z*(N = 32) = −1.72, *p* = 0.085 ^†^, *r* = 0.30).

### 2.5. Positive/Negative Affectivity and Sleep Quality

In order to examine whether evening PA and NA predicted subjective sleep quality, a linear mixed-effects model was carried out. This model encompassed a random factor to consider the within-subject comparisons. The statistical analysis revealed that evening PA (*b* = −0.37, *S.E._b._* = 0.11, *df* = 41.12, *t* = −3.36, *p* = 0.002) but not NA (*b* = 0.043, *S.E._b._* = 0.10, *df* = 81.52, *t* = 0.42, *p* = 0.675) predicted next-day self-rated sleep quality, i.e., higher evening PA predicted higher subjective sleep quality. Furthermore, evening PA and NA did not predict any of the predefined objective sleep quality indices (i.e., SEFF, SOL, awakening index and WASO). Exploratory data analyses on evening PA/NA revealed by trend a main effect for condition concerning evening NA (*χ*^2^(2) = 5.23, *p* = 0.073, *W* = 0.08). Post-hoc comparisons showed that NA in the evening was lower after reading on a smartphone without (*z*(N = 33) = −2.76, *p* = 0.006, *r* = 0.48) or with (*z*(N = 33) = −2.38, *p* = 0.019, *r* = 0.41) a filter as compared to reading a book. For evening PA we also found, by trend, a main effect for condition (*χ*^2^(2) = 5.55, *p* = 0.062, *W* = 0.08). Post-hoc pairwise comparisons showed that PA was higher in the “no filter” (*z*(N = 33) = −2.62, *p* = 0.009, *r* = 0.46) condition as well as by trend higher in the “filter” (*z*(N = 33) = −1.75, *p* = 0.081 ^†^, *r* = 0.30) condition compared to the “book” condition.

### 2.6. Interrelations between Sleep, Circadian and Affectivity Parameters

Furthermore, we were interested in whether circadian parameters (i.e., melatonin AUCi from pre-light exposure until bedtime) and self-rated sleepiness (i.e., increase in sleepiness from pre-light exposure until bedtime), together with evening positive affectivity predicted sleep quality (i.e., awakening index, SWA and SWS in the first night quarter) and circadian (i.e., DPG) parameters, which were affected by the light exposure. We performed linear mixed-effects models in order to test for these relationships. Statistical analyses indicated that none of the tested variables significantly predicted either the awakening index, the amount of SWS or SWA in the first night quarter, or the DPG at 03:00 (i.e., time point when differences emerged between the “no filter” and “book” condition and between the “filter” and “book” condition). Additionally, we tested whether the same variables predicted subjective sleep quality. Evening PA (*b* = −0.38, *S.E._b_* = 0.10, *df* = 42.67, *t* = −3.59, *p* = 0.001) emerged again as a significant predictor and the increase in sleepiness was found to explain additional variance (*b* = −0.22, *S.E._b._* = 0.10, *df* = 74.53, *t* = −2.19, *p* = 0.032), i.e., higher evening PA and a higher increase in sleepiness predicted better subjective sleep quality. AUCi melatonin (from pre-light exposure until bedtime) closely failed to predict subjective sleep quality by trend (*b* = 0.17, *S.E._b_* = 0.10, *df* = 55.21, *t* = 1.64, *p* = 0.108). Regarding the CAR, we found that a lower awakening index (*b* = −0.23, *S.E._b_* = 0.12, *df* = 41.66, *t* = −1.94, *p* = 0.059) predicted by trend a higher cortisol AUCi in the morning. The DPG (at 03:00) did not explain additional variance.

## 3. Discussion

This study investigated the impact of evening short-wavelength light on sleep and circadian rhythmicity. We assessed light-induced changes due to evening smartphone use (with and without filter) in comparison to reading a book under dim light conditions. Additionally, we investigated whether a blue-light filter is able to attenuate light-induced effects. Furthermore, the impact of positive and negative affective states in the evening on subjective and objective sleep quality was assessed. The results provide evidence that short-wavelength light can affect objective sleep as well as circadian parameters (i.e., distal-proximal gradient, melatonin and cortisol awakening response). However, light exposure did not impair subjective sleepiness in the evening. Interestingly, we found that a blue-light filter partially reduced some of these negative effects. Besides external light cues, we demonstrated that in general higher positive, but not negative affectivity in the evening predicted better subjective sleep quality in the subsequent night, although this was not true for measurements of objective sleep quality.

Regarding subjective sleepiness (Figure 1), no differences were found during the evening between the light conditions. However, subjects were less tired at wake-up and, by trend, less tired 30 min later after reading on a smartphone with a filter compared to reading a book. These findings contradict the results of Grønli and colleagues [27], who reported a reduction in sleepiness after reading for 30 min on an iPad compared to reading on printed material, whereas in the morning subjects were less tired after reading from a book. These differences might be related to the fact that the participants in Grønli’s study read a story immediately before turning the lights off, i.e., later in the evening than in our design, when the propensity to fall asleep is generally higher and therefore might be more strongly affected by light cues. However, this could not account for our finding of a trend for lower sleepiness in the morning after reading on a smartphone with a filter compared to reading a book. Cajochen and colleagues [34] reported a circadian but no homeostatic modulation of subjective sleepiness. In the context of our results, this indicates a low responsiveness of the experienced sleepiness in the evening to short-wavelength light emitted by a smartphone display. Furthermore, it emphasizes the divergence between subjective and objective (i.e., hormonal) measurements, as we did find changes in melatonin secretion during the evening despite the lack of effects on subjective sleepiness.

The raw melatonin concentration differed only slightly between the light conditions during the evening. However, after 30 min of light exposure the melatonin concentration was reduced after reading on a smartphone with a filter compared to when the filter was switched off. At bedtime, the raw melatonin level was reduced after reading on a smartphone with a filter compared to reading a book. After baseline correction of our data (i.e., values from pre-light exposure were subtracted from respective later time points), we found, by trend, an attenuated melatonin increase after reading on a smartphone with a filter compared to reading a book 30 min after light exposure and a significantly lower increase 60 min after light exposure as well as at bedtime. Further, the melatonin increase was now also attenuated after reading on a smartphone without a filter compared to reading a book, starting 60 min after light exposure and persisting until bedtime. After 90 min of light exposure, the melatonin increase additionally differed between the smartphone conditions, with a lower increase when reading on a smartphone without a filter as compared to when the filter was switched on (Figure 3). In line with earlier findings [21] these results show a melatonin suppression after reading on a smartphone with and without a filter compared to reading a book. Moreover, our findings indicate a suppressive effect of short-wavelength light exposure compared to exposure to light with longer wavelengths (i.e., by using a filter), at least when the data is analyzed relative to baseline. This effect was expressed by a lower melatonin increase without a filter compared to with a filter, not directly after the exposure started, but after 90 min of light exposure. This emphasizes the high sensitivity of ipRGCs to short-wavelength light, leading to lower melatonin secretion as reported in previous studies [21,35]. Furthermore, it demonstrates an attenuating effect of a filter, at least on melatonin levels as hypothesized by Phelps [36] and in agreement with results from van der Lely and colleagues [16]. The melatonin AUCi was significantly smaller after reading on a smartphone without a filter and, by trend, smaller when reading with a filter compared to reading a book. This further indicates that the pure melatonin increase (i.e., baseline corrected) throughout the evening was attenuated by short- and longer wavelength light. As the melatonin levels between the smartphone conditions differed only at one time point, it is evident that the total evening melatonin increase (i.e., melatonin AUCi) did not differ between the two conditions. However, this raises the question as to whether the melatonin levels would further differ during sleep. Melatonin concentration is closely related to the body temperature, i.e., higher nightly melatonin concentration relates to a higher DPG [37]. Therefore, we utilized the DPG during the night to obtain at least an estimate for longer lasting circadian effects. We found a significantly lower DPG (indicating a lower melatonin secretion) after reading on a smartphone without a filter compared to reading a book between 3:00 a.m. and 4:30 a.m. (Figure 4). After using a filter compared to reading on printed material, the DPG was by trend lower at 3:00 a.m. These results indicate less heat loss after using a smartphone without a filter and partially persisting under usage of a filter function. Contrary to Cajochen and colleagues [21], we did not find differences in the body temperature during and directly after the short-wavelength light exposure, which they interpreted as an indicator for a phase delay shift. Nevertheless, our results of a reduced DPG during the night together with the acute melatonin suppression effects could indicate the beginning of a circadian phase shift. However, for a more elaborated analysis regarding the actual presence of such a circadian phase delay, a longer assessment period of the participants’ melatonin concentration and body temperature would have been required. Comparing the acute effects against a baseline night and evaluating light-induced changes in the evening after the experimental night, as Kim and colleagues did in their study [38], would have been important for a well-founded interpretation regarding a potential circadian phase delay. Additionally, Cajochen and colleagues [21] assessed differences in the responsiveness to light exposure by measuring DPG and core body temperature and found that changes in temperature regarding short-wavelength compared to longer-wavelength light exposure were only reflected in the core body temperature, whereas the DPG only differed between no light exposure and light exposure. They proposed that the DPG lacks the power to reflect slight circadian changes, which would be in line with our findings of no significant differences between the smartphone conditions. Moreover, the insensitivity to detect slight changes with the DPG together with the fact that our participants changed their seating position from an upright position to a more relaxing position during the light exposure (from a standard chair into an armchair), which has been shown to induce thermoregulatory changes [20], could account for our finding of no differences in the DPG during the evening between conditions.

Besides general light-induced circadian changes in the evening and during the night, we also found differences between the conditions in the morning. Cortisol concentration at awakening was lower after reading a book compared to reading on a smartphone without a filter. This pattern changed 30 min later, i.e., cortisol concentration was, by trend, higher after reading a book compared to both smartphone conditions. Accordingly, the CAR (i.e., area under the curve with respect to the increase (AUCi) in cortisol) was reduced after reading on a smartphone without a filter compared to reading on a smartphone with a filter and compared to reading on printed material (Figure 2). It has been shown that a higher CAR relates to a lower nightly cortisol level [24] and is time-bound to the process of the transition from sleep to wakefulness, but late-night cortisol secretion is proposed to be under circadian modulation. It is therefore probable that the nightly cortisol level already increased earlier or steeper, respectively, after reading on a smartphone without a filter, which resulted in a lower CAR. Indeed, exploratory analyses showed that a more fragmented sleep (i.e., higher number of awakenings per hour) predicted a lower CAR. Furthermore, objectively assessed hormonal differences partially differed from the subjective measure of sleepiness in the morning, as we found lower sleepiness at awakening to be reflected in a higher cortisol level after reading on a smartphone with a filter compared to reading a book. However, 30 min later subjective sleepiness was, by trend, still lower after reading on a smartphone with a filter compared to reading a book, although levels of cortisol concentration showed an opposite pattern.

Regarding our sleep analyses and in line with earlier findings [27], the amount of SWS was significantly reduced in the first night quarter after reading on a smartphone without a filter compared to with a filter and as compared to reading a book. This pattern changed in the second night quarter. Herein, subjects spent significantly less time in SWS after reading on a smartphone with a filter compared to when the filter was switched off and participants spent by trend less time in SWS after using a filter compared to reading a book (Figure 5). These results indicate a reduction in SWS after short-wavelength light exposure in the first night quarter, but also a delayed SWS reduction due to usage of a filter that emerged in the second night quarter. However, the amount of SWS did not differ between conditions considering the whole night (Table 1), which indicates that differences regarding time spent in SWS faded away overnight.

Supporting earlier findings [27,39] showing a reduction in SWA after short-wavelength light exposure, we found that SWA was significantly reduced in the first night quarter after reading on a smartphone without a filter compared to when the filter was switched on and compared to reading on printed material (Figure 6). Contrary to Muench and colleagues [7] we found no, exploratively assessed, rebound effect (i.e., heightened SWA) in the third night quarter but rather a statistical trend for a further reduction of SWA after reading on a smartphone without a filter compared to reading a book. Accordingly, with regard to the whole night, SWA was, by trend, reduced after reading on a smartphone without a filter compared to reading a book. Furthermore, SWA was significantly reduced after reading on a smartphone without using a filter compared to when a filter was used. In the context of the two-process model of sleep regulation, SWA reflects the amount of prior sleep pressure and is a marker for sleep homeostasis [40]. Studies using forced desynchrony protocols emphasize the relatively low control of the SCN over SWA (for a review see Dijk [41]). Shedding light on the dependency of SWA from circadian rhythm and sleep homeostasis, Lazar and colleagues [42] tested the impact of sleep- and circadian-dependent modulations on slow waves by means of a forced desynchrony protocol (i.e., participants are exposed to an artificially prolonged or shortened day-night rhythm). They reported that all tested parameters relating to slow waves (e.g., SWA) reacted to circadian modulations, but not to the same extent. Maximum slope and mean duration of slow waves showed the largest response to circadian modulation (i.e., circadian rhythmicity), which was comparable to the influence of sleep-dependent effects on maximum slope and mean duration. Our results corroborate and extend these findings by showing a reduction of SWA in the first night quarter and over the whole night after short-wavelength light exposure indicating a clear circadian modulation. However, looking closer at methodological differences, previous studies analyzed delta power separately for the lower (0.75–1.99 Hz) and upper (2–4 Hz) delta band [27,39]. This method might be more sensitive to detect power changes within the frequency band. Furthermore, in the present study no dark adaptation phase (where participants stay in a dark room for around 2 h) preceded the light exposure. It is obvious that this condition deviates strongly from natural behavior, but is supposed to boost light-induced effects and to standardize the individual light history across subjects [43]. As we wanted to emphasize the impact of short-wavelength light exposure under ecological valid and real-life conditions, we did not implement a dark-adaptation period preceding the light exposure, which might be the main explanation for even more pronounced effects on sleep architecture in earlier studies.

Exploratory data analysis on sleep architecture showed a reduced total sleep time and sleep efficiency along with a higher total wake time after initial sleep onset and a stronger sleep fragmentation after reading on a smartphone with and without a filter as compared to reading a book. According to Lazar and colleagues [42], sleep efficiency was highest when subjects were asked to sleep according to their melatonin rhythm. In the present study, the melatonin AUCi was highest in the book compared to both smartphone conditions, therefore participants experienced a misalignment between the forced bedtime and their melatonin rhythm after using a smartphone with or without filter, which may have lowered the sleep efficiency and related parameters. However, it is important to note that descriptive differences in the mentioned parameters were small. Overall, we were only able to detect some partially protective effects of the blue-light filter regarding sleep in terms of slow wave activity but not with regard to the general sleep architecture, which is in line with and extends recent research from Duraccio and colleagues who also were not able to detect an effect on sleep architecture recorded with wrist actigraphy [44].

Besides light-induced changes in sleep and circadian rhythm, we were interested in the impact of positive (PA) and negative affectivity (NA) on subjective and objective sleep quality. In line with earlier findings [31], we provide evidence that PA but not NA predicted the subsequent subjective sleep quality but neither PA nor NA predicted any of the tested objective sleep quality indices (i.e., sleep onset latency, sleep efficiency, wake after sleep onset and awakening index). Accordingly, the present results suggest that the intensity of NA is not sufficient to affect subjective sleep quality, rather the presence of positive emotional states is crucial to influence sleep positively [45,46]. Supporting this assumption by approaching the topic from the other side, individuals scoring low on PA and high on NA scales reported the highest insomnia severity [46]. This corroborates the idea that NA is preferable to be seen as catalyst for negative influences on sleep quality caused by low PA. Here it has to be considered that we only included mentally healthy subjects who in general show rather low NA. However, it is important to note that the PANAS is indeed normed for a healthy population [47,48]. Another study by Garcia and colleagues [49] categorized individuals regarding their affectivity patterns and classified individuals with high PA and low NA as “self-fulfilling”. These individuals experienced the highest psychological well-being (e.g., self-acceptance) as compared to other groups with different affectivity patterns. Taking these results into account, high PA may generally relate to a higher positive attitude towards life and to more encouraging thoughts in the evening [50], thus leading probably to less rumination and promotion of good sleep quality. Taken together, individuals with high PA tend to concentrate more on positive aspects of the current situation. Supposedly, this positive view also accounts for higher subjective sleep quality ratings in the morning. Extending these findings by gaining information about the influence of affective states on objective sleep quality, measurements of wake after sleep onset, awakening index, sleep onset latency and sleep efficiency were examined. Neither PA nor NA significantly predicted any of these measurements. This is pointing towards the hypothesis that high positive affectivity influences an individual’s perspective concerning self-related ratings (e.g., regarding sleep quality), probably accounting for the present deviation between objective and subjective sleep quality measures. This finding is highly relevant regarding psychotherapy for insomnia patients, especially for those with misperception insomnia (i.e., patients who perceive their sleep quality as bad, but without abnormalities in the PSG).

In addition to our main analyses, we examined whether pre-defined variables in the evening (i.e., PA, melatonin AUCi and subjective sleepiness increase) can predict light-affected sleep (i.e., awakening index, early SWA and SWS) and circadian parameters during the night (i.e., DPG). We found that none of the tested variables emerged as a significant predictor. However, analyses with the same factors regarding subjective sleep quality showed that, besides a higher PA, the increase in sleepiness over the evening predicted better subjective sleep quality. In the context of a “social jetlag” (i.e., the discrepancy between the biological and the social clock), subjective sleep quality was better when subjects reported less severe social jetlag symptoms [51]. The subjects in our study that reported a higher increase in sleepiness during the evening went to bed at a time when they felt biologically ready to sleep (i.e., in accordance with the social clock) and subsequently rated their subjective sleep quality better in the next morning. Further exploratory analyses indicated that a lower average number of awakenings per hour predicted a higher CAR. This is well in line with and extends earlier findings of a relationship between spontaneous nightly awakenings and heightened nightly cortisol secretion [52], which in turn relates to a lower CAR [24]. Additional exploratory analyses showed that PA was significantly higher after reading on a smartphone with a filter and by trend higher after reading on a smartphone without a filter compared to reading on printed material. In contrast, NA was lower after reading on a smartphone with and without a filter compared to reading a book. At a first glance this result might appear unexpected, as higher electronic media use in the evening was associated with higher depressive symptoms mediated by sleep difficulties in an earlier study [4]. However, in contrast to the study from Lemola and colleagues [4], who examined the individually selected electronic media content, in the present experiment all participants were exposed to the same content (i.e., the same stories) while reading on a smartphone or from a book. Thus, the pure light-effect was examined and related to lowered states of NA and heightened states of PA. Results from Partonen and Lönnqvist [53] support these findings by showing vitality enhancing and distress lowering effects of frequent self-applied bright-light exposure during winter in healthy individuals. Further evidence from clinical samples supports the applicability of bright-light exposure as a treatment for affective disorders (for a review see Wirz-Justice and colleagues [54]). Indeed, this treatment led to mood enhancements in patients suffering from major depression [55]. Regarding underlying neuronal pathways, first findings in animal models revealed a thalamic region (i.e., perihabenula nucleus) as a mediator between light stimuli and affectivity changes [56].

In sum, our results support the notion that artificial light exposure does not only affect sleep and circadian rhythms, but also affective states in healthy subjects in opposite ways; light had negative influences on sleep and circadian rhythmicity, but mood ameliorating effects. Influencing our mood in the evening by short-wavelength light can positively affect our perceived sleep quality. At the same time, the light can negatively affect subsequent objective sleep and circadian parameters. This should imply, on the one hand, that in the evening alternative methods are highly needed to enhance evening positive affectivity. On the other hand, it implies that possible negative long-term consequences (i.e., daytime sleepiness, attentional issues) due to effects on the sleep and circadian rhythmicity might remain unrevealed, as individuals perceive their sleep quality as good.

### Limitations

The study was carried out over the course of more than a year and therefore during different seasons. Therefore, it might lack comparability to data, which were recorded only during one season, as results were not controlled for individuals’ light exposure prior to laboratory nights. However, light exposure, especially in the morning, is also capable of influencing the circadian rhythm [57,58]. Besides this seasonal aspect, we only included men in our study. This decision was driven by our own recent findings suggesting that menstrual cycle-dependent oscillations in sex hormones affect sleep and cognition [59]. Additionally, Baker and Drivers [60] reported hormonal cycle-dependent changes in sleep architecture in naturally cycling women. Although men are less subject to monthly hormonal fluctuations, melatonin [61] and cortisol [62] secretion are also affected by seasonal changes in men. Besides these issues of our study protocol and our sample, studies examining light influences differ significantly regarding the applied display size and type (e.g., LED or LCD screens and computers, e-book readers or tablets/smartphones), brightness and size. Furthermore, Chellappa [3] concludes that circadian photosensitivity is subject to interindividual differences, i.e., the response to artificial light in the evening, such as changes of circadian parameters, varies largely across individuals. An example for an individual trait responsible for the high variance in light-susceptibility across individuals might be eye pigmentation, as one study reported stronger melatonin suppression in subjects with lighter eye colors (i.e., blue, green or light-brown iris compared to dark brown iris) [63]. Thus, we cannot rule out that we included more high- than low-responders or the other way around. Besides general interindividual differences in sensitivity to light, it is additionally affected by age [28]. Younger adults showed much stronger light-induced changes in circadian (i.e., endogenous melatonin secretion) and sleep parameters (i.e., frontal SWA), subjective sleepiness and attention in contrast to older adults. This might not have been an issue regarding the present results, as our study sample consists of a very homogeneous age group, but rather an issue concerning comparability between studies investigating light exposure with subjects of different age groups. Future studies should therefore address this issue regarding general interindividual and age-related differences in light-responsiveness to gain more insight into the interaction between the daily present artificial light consumption and our inner clock.

## 4. Materials and Methods

### 4.1. Participants

33 healthy male subjects (mean age: 21.70, standard deviation: 1.91, range: 18–25 years) were recruited and examined at the University of Salzburg between October 2019 and December 2020. The present results extend our already published preliminary data of a subset of 14 participants [64]. All subjects were free of medication, non-smokers and reported no history of drug abuse, night-shift working, neurological or psychiatric disease. Further, they were right-handed, showed no above average caffeine consumption (i.e., >3 cups of coffee, or >1 energy drink per day) and were not extreme chronotypes (defined as subjects with raw sum-scores below 31 or above 69) according to the German version of the morningness-eveningness questionnaire [65]. For the entire study period sleep habits were monitored with wrist actigraphy (Cambridge Neurotechnology Actiwatch^®^, CamNTech Ltd., Cambridge, England) and sleep diaries (“morning/evening protocol”; adapted version of Saletu and colleagues [66]) to assure a regular sleep-wake cycle. Participants were remunerated with either 100 Euros and 16 h of course credit for participation in scientific studies or with 50 Euros and 24 h of course credit. All participants provided written informed consent. The study was approved by the local ethics committee and performed in accordance with the latest version of the Declaration of Helsinki (2013).

### 4.2. Procedure

**Figure 7:**
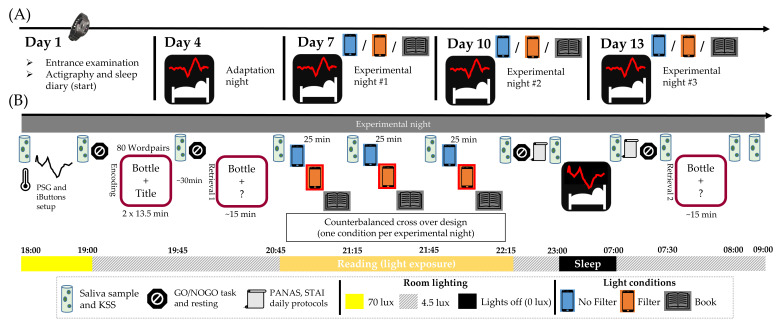
Study design [64]. (**A**) Overview of the general procedure, which covered a period of 13 days, including an entrance examination, one adaptation night and three experimental nights. (**B**) Detailed illustration of the study protocol in the experimental night. During the whole study period, participants’ sleep-wake rhythm was monitored by means of wrist actigraphy and sleep diary.

As depicted in Figure 7, the study covered a period of 13 days per participant. Before the subjects were admitted to the study, they filled in an online entrance questionnaire (LimeSurvey: An Open Source survey tool, LimeSurvey GmbH, Hamburg, Germany). On the first day of the study protocol, participants came to the sleep laboratory for an entrance examination to receive all information for their participation. Additionally, they provided data about their daily activities by using the app Time Use Diary (version 1.0.1, (Google Commerce Ltd., Dublin, Ireland)) [67] and about their daily smartphone use by using the app Murmuras (version 2.4.3, (Google Commerce Ltd., Dublin, Ireland)) [68]. Both apps were installed on their private smartphones. On day four, an adaptation night took place. Therefore, participants arrived at the laboratory at 9:00 p.m. After polysomnography (PSG) and temperature buttons montage, a resting condition followed by a GO/NOGO task (i.e., an auditory response inhibition and attention test [69]) was performed. Before and after bedtime positive (PA) and negative (NA) affectivity (PANAS [48]), as well as state anxiety (STAI-S [70]) were evaluated. Participants went to bed around 11 pm and were woken up exactly 8 h later. In the morning, the resting condition as well as the GO/NOGO task were carried out again. Before leaving the laboratory, subjects had to perform an intelligence test (i.e., Advanced Progressive Matrices [71]). On day seven, ten and thirteen, the experimental nights took place and participants arrived at 6:00 p.m. at the laboratory. After PSG and the temperature buttons montage, room light (Emilum GmbH, Oberalm, Austria) intensity was adjusted to 4.5 photopic lux until the end of the experiment, except for the 8 h of sleep when all lights were turned off (0 photopic lux). Before the light exposure, participants performed a declarative word-pair learning task (adapted from Schabus and colleagues [72]) consisting of two encoding blocks, each comprising 80 word pairs, followed by a cued retrieval session. Afterwards, the reading session (light exposure) took place, which consisted of three 25 min blocks where participants read the three following stories in a randomized order: (1) “555 populäre Irrtümer: Warum Angela Merkel eigentlich ein Wessi ist, man Eier nicht abschrecken muss und Erdnüsse keine Nüsse sind” [73], (2) “Die Känguru Chroniken: Ansichten eines vorlauten Beuteltiers” [74] and (3) “13 gegen das Sommerloch: 13 Autoren-13 Geschichten-13 x Lesespaß” [75]. The stories were either presented on a standardized smartphone (Samsung Galaxy A50 enterprise edition; Samsung Electronics, Seoul, Korea) (1) without blue-light filter (i.e., filter was switched-off), (2) with blue-light filter (i.e., filter was switched-on) or in the form of a (3) printed book.

The light conditions were counterbalanced across the three experimental nights. Light characteristics were calculated using luox [76], a validated web-based open-source platform for spectral calculations [77]. Light characteristics differed between the smartphone conditions in the following way: the display showed a melanopic radiance of 286.77 mW × m^−2^ × sr in the “no filter” condition and a melanopic radiance of 114.20 mW × m^−2^ × sr in the “filter” condition. The correlated color temperature was at 8298 K in the “no filter” and at 3032 K in the “filter” condition. Light characteristics were measured (height from floor to eye-level: 87 cm, distance from eyes to stimulus: 37 cm) using a spectrometer (JETI spectraval 1501; JETI Technische Instrumente GmbH, Jena, Germany). In the “no filter” and “filter” conditions the stories were presented as e-book versions on the smartphone. In the “book” condition, the stories were printed according to the format and text size of the e-book versions and presented as a ring folder to assure comparability between conditions. After each 25 min block of reading, subjects were verbally asked four to six questions about the story to assure compliance with the study protocol. Whenever participants used the lab phone or left the room (i.e., during six hourly toilet breaks of approximately 5 min), they wore orange-dyed glasses (Uvex Skyper Blue-Light Blocking Computer Glasses; Honeywell, Charlotte, NC, USA) to avoid unstandardized short-wavelength light exposure. Participants went to bed around 11 pm. Before and after the night they completed the PANAS, STAI-S and the evening/morning protocol. In the morning, the second cued retrieval session of the word-pair task took place. Participants left the laboratory around 9 am. Saliva samples were collected at 12 fixed time points during each experimental night (cf., respective time points depicted in Figure 7B). Concurrently with the saliva samples, participants rated their subjective sleepiness on the Karolinska Sleepiness Scale (KSS) [78]. Furthermore, participants performed a GO/NOGO task four times, which was always preceded by a resting condition. During the laboratory stays, the standardized lab phone was provided to the subjects to fill in the morning and evening protocols.

### 4.3. Subjective Measurements

Situational, self-reported sleepiness was measured by means of the German version of the KSS. The instrument contains one item (“How sleepy do you feel at the moment?”) with a 10-point Likert scale (1 = extremely alert; 5 = neither alert nor sleepy; 10 = extremely sleepy, can’t keep awake). The KSS was verbally applied at the aforementioned time points.

Self-reported positive (PA) and negative affectivity (NA) was measured using the German version of the PANAS. The questionnaire comprises two 10-item scales assessing self-reported PA (e.g., active, interested) and NA (e.g., distressed, upset) on a 5-point Likert scale (1 = very slightly or not at all; 5 = very much), whereby an average value for both scales can be calculated.

Self-rated sleep quality was assessed by the morning protocol on a daily basis. The measurement contains seven items comprising questions regarding subjectively experienced sleep quality of the last night (e.g., about difficulties to fall asleep), rated on a 4-point Likert scale ranging from “no” (=1) to “very” (=4). The sum score is calculated with higher values indicating worse self-rated sleep quality. Objective sleep quality was assessed by the following sleep parameters derived from the PSG: sleep onset latency to N2 (SOL in minutes), wake time after sleep onset (WASO in minutes), awakening index (i.e., average number of awakenings per hour) and sleep efficiency (SEFF in %).

### 4.4. Polysomnography and Temperature

The EEG setup comprised 11 gold cup scalp electrodes (Grass Technologies, Astro-Med GmbH, Rodgau, Germany) applied at F3, Fz, F4, C3, Cz, C4, P3, Pz, P4, O1, O2. Fpz served as ground electrode and the signal was re-referenced offline to averaged mastoids (A1 and A2). Additionally, two vertical and two horizontal electrooculography electrodes were placed. Two electromyography electrodes were adjusted at the musculus mentalis. In total, five temperature buttons (iButton DS1922L; Maxim Integrated Products, Inc., San Jose, CA, USA) were attached to the participant’s body. Two were proximally located relative to the body center (i.e., in an infraclavicular position at left and right body sides) and two were distally located relative to the body center (i.e., at right and left ankle positions). One additional button was attached to the head box to record the room temperature. Temperature was continuously measured in 5 min intervals during the whole stay in the laboratory starting at 8 pm.

EEG was recorded using a 32 channel BrainAmp system (Brain Products GmbH, Munich, Germany) with a sampling rate of 500 Hz. Data processing was done using the BrainVision Recorder software (Version 2.11, Brain Products GmbH, 2015). Impedances between the scalp and the electrodes were kept below 10 kΩ. Sleep was automatically staged by the SOMNOLYZER 24 × 7 (Koninklijke Philips N.V.; Eindhoven, The Netherlands) and visually controlled by an expert following the criteria of the American Academy of Sleep Medicine [79]. Due to technical issues (i.e., recording crashed during the night), one participant was excluded from all sleep analyses.

For further analyses of the skin temperature, the distal-proximal gradient (DPG) was computed by subtracting the averaged proximal skin temperature values from the averaged distal skin temperature values at each sampling point. Due to technical issues (i.e., iButtons fell off), three participants were excluded from all DPG analyses. In order to discover relationships between temperature data and measurements of subjective sleepiness as well as melatonin and cortisol, the mean DPG was then calculated in accordance with time points of KSS assessment and saliva samplings + 10 min (i.e., average of three DPG values per sampling point) to avoid overestimation of outliers. The same procedure was followed during sleep, where the mean DPG was calculated as described every 30 min.

### 4.5. Slow Wave Activity Analysis

For each electrode, data were cut into 2700 ms segments (based on recommendations from Cohen [80]) for the whole night and for each night quarter (i.e., 2 h of sleep). Power per frequency bin was calculated by using Welch’s Method of the Fast Fourier Transform (FFT) with a sliding Hamming window function. Slow wave activity (SWA) was defined as the average amount of power (µV^2^) within the delta frequency range (0.75–4.5 Hz). The results were then averaged over frontal (F3, Fz, F4), central (C3, Cz, C4), parietal (P3, Pz, P4) and occipital (O1, O2) electrodes.

### 4.6. Cortisol and MelatoniN Analysis

All saliva samples were stored at −20 °C until they were centrifuged twice—first for 15 min and then again for 10 min—in order to remove solid particles and mucus. Saliva samples were then refrozen and restored at −20 °C. Salivary cortisol was analyzed using the DeMediTec cortisol free in saliva ELISA (DeMediTec Diagnostics GmbH, Kiel, Germany). We evaluated six samples regarding the cortisol concentration, i.e., before light exposure, after 90 min of light exposure, at bedtime, directly after awakening, as well as 30 min and 60 min after participants woke up. The cortisol awakening response (CAR) was calculated as the area under the curve (AUC) with respect to the increase over the first 60 min after awakening (AUCi [81]). In general, the AUC is either computed with respect to the ground (AUCg), assessing the total cortisol output, or with respect to the increase (AUCi), which considers a baseline measurement and estimates state-dependent changes over time [82]. Hence, we were interested in the AUCi in order to assess light-induced changes over time. Additionally, the absolute cortisol increase within 30 min after awakening was computed (i.e., the difference between awakening and 30 min afterwards). Salivary melatonin was analyzed using SALIMETRICS salivary melatonin enzyme immunoassay kits (Salimetrics Europe, Suffolk, UK). Regarding melatonin concentration, we evaluated six saliva samples, i.e., before light exposure, after 30 min, 60 min and 90 min of light exposure, at bedtime and directly after participants woke up. In order to examine the melatonin increase in the evening, the AUCi was calculated for the melatonin increase in the evening from pre-light exposure [20:45] until bedtime [23:00].

Each saliva sample was analyzed twice. Whenever the coefficient of variation between these two samples exceeded the 25% threshold, samples were analyzed again. Non-detectable concentrations, i.e., concentrations lower/higher than the lowest/highest standard were set at the threshold of the lowest/highest measurable concentration. Missing values (e.g., due to missing samples) were replaced by the interpolated value (i.e., mean of the values of the following and the previous assessment time point). In case of a missing value right before sleep or directly after awakening, the sample mean was used.

### 4.7. Statistical Analyses

For statistical analyses SPSS (Version 27.0.0.0; IBM Corp., Armonk, NY, United States, 2020), R-Studio (Version 1.4.1103) and Microsoft Excel (Version 16.30, Microsoft^®^ Excel for Mac, Microsoft, Redmond, WA, United States, 2019) were used. Ggplot2 [83] was utilized for data visualization. 95% confidence intervals around the mean for a within-subject design were calculated with the Rmisc package [84].

## Figures and Tables

**Table 1:** Whole night sleep architecture for all conditions (median and interquartile range). Non-parametric Friedman tests were conducted (N = 32).

	No Filter	Filter	Book	χ^2^	*p*
TIB (min)	480.50 (0.38)	480.50 (0.50)	480.50 (0.50)	3.34	0.188
TST (min)	463.75 (34.63)	463.75 (25.50)	468.50 (17.25)	16.75	<0.001 **
SEFF (%)	97.03 (6.53)	96.57 (5.44)	97.50 (3.15)	11.67	0.003 *
SOL N2 (min)	13.00 (8.13)	10.75 (9.88)	11.00 (10.13)	4.12	0.128
N1 (%)	12.77 (7.38)	11.34 (6.11)	10.74 (6.73)	0.44	0.804
N2 (%)	38.80 (9.36)	39.38 (8.99)	39.66 (7.56)	0.19	0.911
N3 (%)	27.72 (12.21)	28.34 (9.42)	28.22 (8.85)	0.49	0.783
REM (%)	19.58 (7.47)	19.97 (7.27)	20.67 (7.22)	0.81	0.666
WASO (min)	10.25 (20.75)	11.00 (17.38)	7.25 (9.88)	6.50	0.039 *
Awakening Index	1.49 (2.10)	1.35 (1.08)	1.17 (1.45)	6.49	0.039 *

Note. TIB = Time spent in bed, TST = Total sleep time, SEFF = Sleep efficiency; SOL to N2 = Sleep onset latency to N2; WASO = Wake time after sleep onset; Awakening Index = Number of awakening per hour. **: *p* < 0.001, *: *p* ≤ 0.05.

## Supplementary Materials

The following are available online at https://www.mdpi.com/article/10.3390/clockssleep3040040/s1.
