# Supplementary material for: How Smart Is It to Go to Bed with the Phone? The Impact of Short-Wavelength Light and Affective States on Sleep and Circadian Rhythms
Source: Clocks Sleep. 2021 Oct 28;3(4):558–80. doi: 10.3390/clockssleep3040040 (PMC8628671; doi:10.3390/clockssleep3040040)
Supplement: Supplementary file 1 [file clockssleep-03-00040-s001.zip › Table S1.pdf]

Table S1. SWA overview (median and interquartile range). Non-parametric Friedman tests were carried out ($N = 32$).

		No Filter	Filter	Book	X^2	p
Night quarter 1	Frontal	15.43 (13.90)	16.46 (14.23)	16.71 (13.64)	7.31	0.026*
	Central	10.45 (8.55)	11.43 (6.38)	11.39 (7.52)	9.19	0.010*
	Parietal	7.79 (7.35)	8.91 (4.40)	8.62 (6.46)	9.81	0.007*
	Occipital	3.28 (3.49)	4.65 (3.39)	3.97 (4.25)	2.31	0.315
Night quarter 2	Frontal	9.43 (6.56)	8.61 (7.00)	9.61 (4.80)	0.81	0.666
	Central	6.83 (4.58)	6.12 (3.68)	6.16(2.96)	1.31	0.519
	Parietal	5.02 (4.53)	4.81 (3.14)	4.67 (2.59)	1.75	0.417
	Occipital	2.62 (2.38)	2.95 (2.44)	2.83 (1.64)	0.75	0.687
Night quarter 3	Frontal	7.21 (4.38)	6.64 (3.40)	7.39 (4.34)	1.75	0.417
	Central	5.02 (2.89)	4.88 (2.50)	5.37 (2.62)	3.94	0.140
	Parietal	3.82 (2.29)	3.49 (2.09)	4.09 (1.81)	3.06	0.216
	Occipital	2.12 (1.97)	2.22 (1.63)	2.66 (1.60)	3.06	0.216
Night quarter 4	Frontal	5.38 (2.81)	5.01 (4.51)	4.70 (3.16)	0.44	0.804
	Central	3.95 (2.30)	3.74 (2.32)	3.72 (2.70)	0.06	0.969
	Parietal	3.07 (1.83)	3.17 (1.98)	3.07 (2.39)	0.81	0.666
	Occipital	1.61 (1.22)	1.85 (1.87)	1.56 (1.67)	1.94	0.380
Whole night	Frontal	10.27 (5.62)	10.73 (5.90)	10.02 (4.05)	10.19	0.006*
	Central	7.05 (4.24)	7.13 (3.31)	7.27 (3.02)	4.00	0.135
	Parietal	5.48 (3.07)	5.55 (2.49)	5.25 (3.08)	7.75	0.021*
	Occipital	2.63 (2.22)	3.11 (2.21)	2.78 (2.48)	1.75	0.417

Note. * = $p \leq 0.05$
