# Supplementary figures and images for: How Smart Is It to Go to Bed with the Phone? The Impact of Short-Wavelength Light and Affective States on Sleep and Circadian Rhythms
Source: Clocks Sleep. 2021 Oct 28;3(4):558–80. doi: 10.3390/clockssleep3040040 (PMC8628671; doi:10.3390/clockssleep3040040)

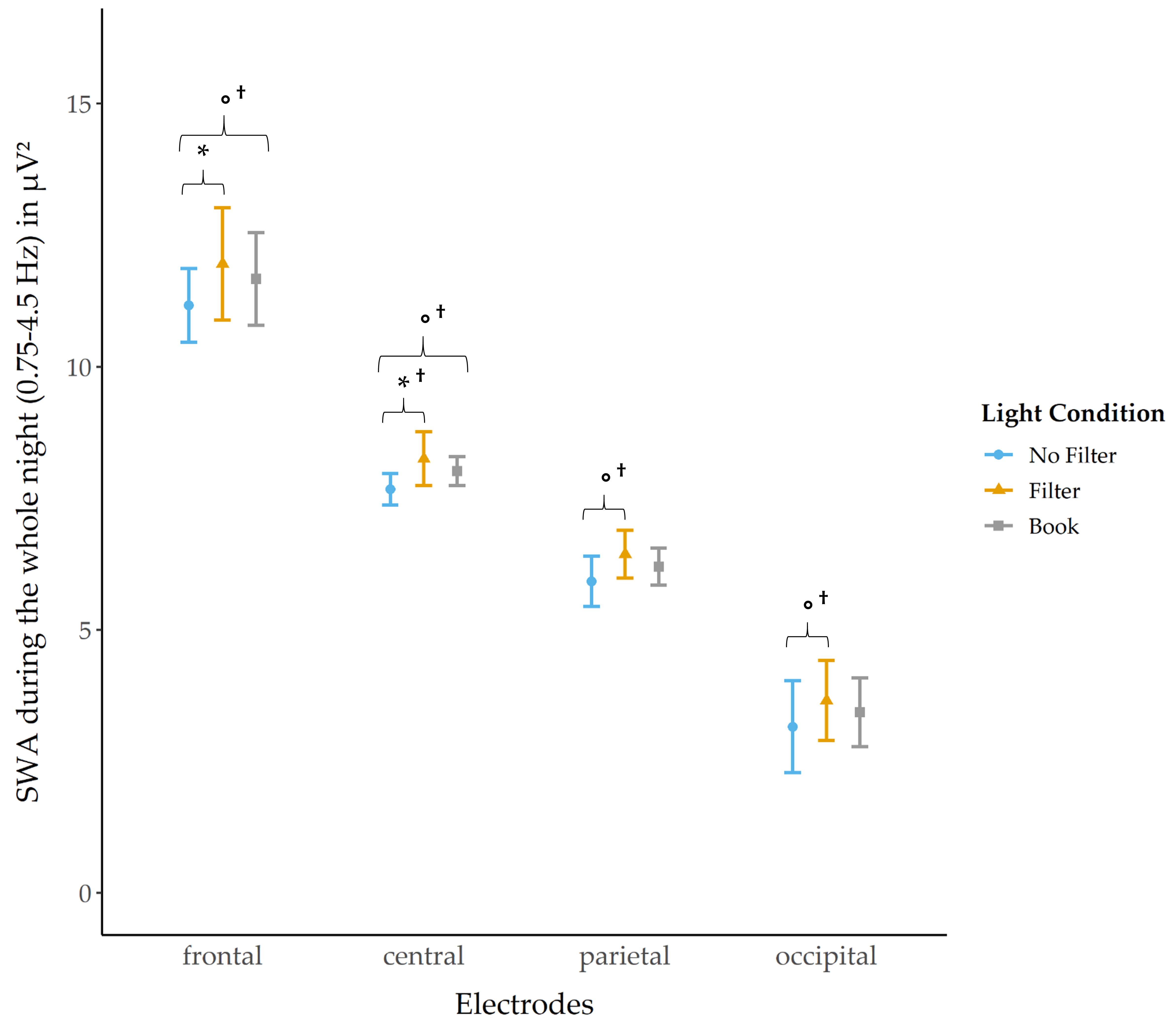

Supplement: Supplementary file 1 [file clockssleep-03-00040-s001.zip › Figure S1.tif]
